# Implementation of the Flexible Assertive Community Treatment (FACT) Model in Norway: eHealth Assessment Study

**DOI:** 10.2196/32220

**Published:** 2022-01-10

**Authors:** Erlend Bønes, Conceição Granja, Terje Solvoll

**Affiliations:** 1 Norwegian Centre for e-Health Research University Hospital of North Norway Tromsø Norway; 2 Faculty of Nursing and Health Sciences Nord University Bodø Norway

**Keywords:** mental health, FACT, electronic health records, eHealth, FACT implementation, EHR, implementation, assessment, model, community, treatment, policy, regulation, infrastructure, literature, challenge, intervention

## Abstract

**Background:**

Flexible Assertive Community Treatment (FACT) is a model for treatment of long-term severe mental disorders. This method has become more widespread in Norway.

**Objective:**

The objective of our study was to examine how the implementation of FACT teams in Norway has been affected by eHealth policy, infrastructure, and regulations. Another objective was to examine existing literature on eHealth interventions and challenges within FACT teams.

**Methods:**

We have examined Norwegian policy regulating mental health services, laws and regulations, eHealth infrastructure, relevant literature on FACT teams, and current implementation of FACT in Norway.

**Results:**

FACT teams are a wanted part of the Norwegian service system, but the current eHealth infrastructure makes sharing of data within teams and levels of health care challenging, even if eHealth regulations allow such sharing. This has been shown to be an issue in the current implementation of FACT teams in Norway. There is little or no existing research on the eHealth challenges facing FACT teams.

**Conclusions:**

Weaknesses in the Norwegian eHealth infrastructure have been a barrier for an easy implementation of FACT teams in Norway. It is difficult to share information between the different levels of health care. We need systems that allow for easy, secure sharing of health information to and between the FACT team members and other involved health care workers.

## Introduction

### Background

In the late 1970s a method was developed in the United States for the treatment of persons with severe mental disorders, the so-called Assertive Community Treatment (ACT) model [1]. An ACT team is a multidisciplinary team that includes case managers, psychiatrists, psychologists, and substance abuse specialists. The ACT model defines the following services to be provided by ACT teams: contact in the community and a holistic approach to care, for example, housing, medication, finances, everyday needs, and continuous coverage, which means that the ACT teams should be available for the patients at all times [1]. The ACT model estimates that the target group is 20% of the persons with a long-term severe mental disorder within a defined area [2]. Data concerning the patients and their pathways are usually displayed on a shared team whiteboard and discussed in daily team meetings. The purpose of the board is to maintain communication within the team, make sure no patients are left out, and make the daily meetings more efficient [3]. In later years, some ACT teams have used electronic whiteboards and videoconferencing to communicate with patients that are able to use this kind of technology [4].

While the ACT model has shown great results in the United States, the results of some European implementations of the model were below expectations [5]. A justification for these results is the different health care models of each country and characteristics of the target group [5]. Additionally, in areas with low population density, the number of persons with severe mental disorders was too low to implement full ACT teams [2]. Because of these challenges, in the early 2000s, a variant of the ACT model intended for rural areas was implemented in the Netherlands, known as Flexible Assertive Community Treatment (FACT) [2]. The main difference between the 2 models is that the FACT model aims at supporting 100% of the persons with severe long-term mental disorders in the area [3], making it better suited for areas with low population density. This implies that many patients do not need continuous intensive follow-up, therefore, FACT teams provide individual case management [2]. As in the ACT model, patients at risk of relapse or readmission receive intensive follow-up from FACT teams. After the FACT model was implemented in the Netherlands, it has spread to a large number of health care teams in Sweden, Norway, and England, despite the lack of conclusive scientific evidence of its effectiveness [6].

### FACT in Norway

In Norway, the government has the responsibility for specialist health care services, which are divided into 4 Regional Health Authorities. The Regional Health Authorities own the hospitals, which are organized as independent health trusts. Specialist mental health services can be provided by both the hospitals and community mental health centers. The responsibility for primary care and local services is assigned to the 356 municipalities. Most Norwegian FACT teams are organized as a cooperation between specialist mental health care and services from 1 or more municipalities [7]. Patients are referred to FACT teams by general practitioners or institutions in the specialist health care.

The ACT/FACT methods are recommended in the *National Health and Hospital Plan 2020-2023* [8] and *Coping With Life: The Government's Strategy for Good Mental Health (2017-2022)* [9]. In line with these recommendations, the Norwegian Directorate of Health has been granting funds for municipalities and health institutions to establish ACT/FACT teams since 2009 [8]. From 2009 to 2013, there were 14 ACT teams established in Norway. In 2013, the first FACT team was established, and in 2020, there were already approximately 70 FACT teams in Norway [10]. This shows the strong focus of the Norwegian Government in establishing FACT teams to support patients with severe mental disorders.

Norway has a population of 5,402,171 and an area of 323,808 km^2^ [11]. Consequently, many persons have long travel distances to the nearest hospital or community mental health center. Therefore, FACT teams in rural areas of Norway typically support several municipalities, leading to distributed FACT teams situated on several locations.

In the context of this paper, we define eHealth as the use of information and communication technology (ICT) to improve efficiency, quality, and security in the health care delivery. The use of eHealth interventions as a solution for geographic and demographic challenges is one of the focus points of the Norwegian eHealth strategy [8]. In this paper, we discuss how the Norwegian eHealth infrastructure and regulations have affected the implementation of the FACT model in the country.

The overall aim of this paper is to conclude on how the implementation of the FACT model in Norway can be improved using eHealth interventions. To this end, we have examined the Norwegian policy regulating mental health services, eHealth regulations, eHealth infrastructure, relevant literature on FACT teams, and knowledge about the current implementation of FACT teams in Norway.

## Methods

### Norwegian Policy Regulating Mental Health Services

To get an overview of the governance of the Norwegian eHealth and mental health sectors we performed a content analysis of policy documents. We searched the governmental sites, The Norwegian government [12], The Norwegian Directorate of Health [13], and The Directorate of eHealth [14], for policy documents within the following main research topics: (1) eHealth, (2) health services delivery, and (3) health services delivery specific for mental health. The content analysis [15] identified the themes of data target by the policy documents. The content analysis included not only currently valid policy documents, but also documents that, even if they are no longer valid, still regulate mental health services.

### Laws and Regulations Governing eHealth

Laws and regulations govern how health care workers do their work, how data are shared in the health care sector, and how ICT systems for health care can operate. For this reason, to understand implementation of ICT systems for FACT teams it is necessary to consider the relevant laws.

To identify relevant laws and regulations, we searched the Norwegian law database [16] for laws and regulations that are currently valid. We also studied the Norm for Information Security and Privacy in the Health and Care Sector (The Norm, from the Norwegian: *Normen for informasjonssikkerhet og personvern i helse- og omsorgstjenesten*), an industry norm that is developed and maintained by organizations and institutions in the Norwegian health care sector.

### eHealth Infrastructure

The eHealth infrastructure has a large impact on what eHealth solutions are available, thus impacting how FACT teams operate. There are several national eHealth services in Norway that are facilitated by the national eHealth infrastructure.

In 2012, the Ministry of Health and Care Services published a governmental white paper—One Citizen–One Journal [17]—with the aim of presenting the goals for ICT development in health care in Norway. In this document the main strategy targeting the eHealth infrastructure indicated that electronic communication should be the way of communicating in the Norwegian health care sector. The Norwegian Health Net (NHN) is an enterprise owned by the Ministry of Health and Care Services, which has the responsibility to manage, operate, and further develop the national eHealth infrastructure.

Among the services provided by the NHN the ones relevant for the FACT team are The Norwegian summary care record (SCR) and the Helsenorge portal [8,18]. The SCR is a collection of health information for patients that is available for all levels of health care in Norway. Helsenorge is a public portal for national digital health services in Norway. The portal contains health-related information for the citizens, personal health information, and various self-service solutions. Examples of self-service solutions are access to the SCR and information about prescriptions and vaccines. We studied official documents issued by the NHN to collect knowledge on important parts of the Norwegian eHealth infrastructure (ie, electronic messages, videoconferencing, and the SCR).

### Relevant Literature on FACT Teams

To get an overview of research already done on eHealth interventions for ACT and FACT teams, we performed literature searches for original papers in the databases PsycINFO and PubMed. We searched PubMed for the large number of articles on eHealth research, and PsycINFO for additional articles with a focus on mental health.

To find relevant articles we made a list of keywords that describe ICT interventions. This list was combined with the search string “assertive community treatment” to find what related to ACT or FACT teams. The full search string is provided below:

“assertive community treatment” AND (ehealth OR e-health OR telemedicine OR telepsychiatry OR ICT OR ehr OR digital OR technology OR video OR whiteboard)

We analyzed the titles and abstracts of the articles that resulted from the search string for inclusion according to the following predefined exclusion criteria: papers that did not report on ICT solutions for ACT or FACT teams, and papers that were not in English. We used the web tool Rayyan [19] for organizing inclusion decisions.

After applying exclusion criteria, we did a full-text analysis on the remaining papers.

### FACT Implementation

The purpose of the Norwegian National Advisory Unit on Concurrent Substance Abuse and Mental Health Disorders (NKROP; from the Norwegian *Nasjonal kompetansetjeneste for samtidig rusmisbruk og psykisk lidelse*) is to run and support various projects and measures with the aim of enhancing health, quality of life, and functional level for persons with concurrent substance abuse and mental health disorders. One part of this purpose is supporting FACT teams with implementation of the FACT model. As part of the unit’s role, NKROP makes available several documents regarding the implementation of the FACT model. We have studied the documents available at the unit’s webpage to get an overview of guidelines for the FACT implementation in Norway. We selected the documents that provided information on the technical implementation of the FACT model.

The FACT Handbook [3] was written by one of the founders of the FACT model and was translated to Norwegian in 2013. This handbook describes the FACT model and how FACT teams work. We studied this document because it has been an important guideline in the practical implementation of FACT teams in Norway. Even though the handbook does not provide explicit information on the technical implementation of the FACT model, we included this document as it provides information that can inform the definition of requirements toward technology.

Norwegian FACT teams were evaluated in a report from 2020 [7]. We also studied this document because it shows many of the experiences of FACT teams.

A new published paper studied how Norwegian FACT teams are integrated into the service system [10]. This paper did not match our inclusion criteria for the literature search, because of a lack of focus on ICT solutions. However, it describes aspects important to the implementation of FACT teams, and thereby it was included in our study.

## Results

### Norwegian Policy Regulating Mental Health Services

The search of the governmental websites identified 6 policy documents relevant for the implementation of FACT teams. The documents were categorized under the main research topics as described below.

#### Main Research Topic 1: eHealth

The governmental white paper *One Citizen–One Journal* (in Norwegian: *Én innbygger – én journal*) [17], published in 2012 by the Ministry of Health and Care Services, aimed at presenting the goals for ICT development in health care in Norway. The main goal of this white paper was to ensure health care workers have easy and secure access to patient and user information; citizens have access to easy and simple digital solutions; and data are available for quality improvement, monitoring, management, and research. The paper also stated that the goal of the government is that all written communication in health care should be electronic. To reach these goals the government wanted to modernize the ICT platform, and work toward a national ICT solution for the whole health and care sector. The white paper also points out challenges of a lack of integration between systems.

#### Main Research Topic 2: Health Services Delivery

*The Coordination Reform* (in Norwegian: *Samhandlingsreformen*), published in 2009 [20], was a report that described a reform of Norwegian health services. The report pointed out 3 primary challenges: services that are poorly coordinated, lack of focus on disease prevention, and an increase in the prevalence of chronic diseases due to an aging population. To meet these challenges, 5 measures were proposed: (1) There should be a stronger focus on user involvement and better patient pathways; (2) Municipalities should have an increased focus on prevention, early intervention, treatment, and follow-up; (3) Economic incentives should be put in place, where municipalities cofinance the specialist health care, and are economically responsible for patients who are ready to be discharged from the hospital; (4) Specialist care should be more focused on specialist tasks. This should be achieved by a better division of work between primary and specialist care. Also, a stronger focus on patient pathways would make patients in need of specialist care get the right treatment; (5) The government should also have a more holistic view on the health care and the patients when prioritizing needs. The report also pointed out that more integrated patient pathways would require new ways of using ICT solutions and emphasized the challenges of ICT systems not communicating well enough. Also, many beds in specialist mental health are used by patients who should have received stronger follow-up from municipalities instead. ACT teams are described as one way of achieving this.

The *National Health and Hospital Plan 2016–2019* (in Norwegian: *Nasjonal helse- og sykehusplan 2016–2019*) [21] presented the governments goals for the development of specialist health care for the period 2016-2019. The overall goals were to focus on patient-centered care; prioritize the field of mental health and addiction; renew, improve, and simplify services; contribute to enough health care workers with the right competences; improve quality and patient safety; improve division of responsibilities and cooperation between hospitals; and improve emergency medicine outside the hospitals. The plan states that the field of mental health and addiction should be prioritized, and that specialist health care should be provided close to where the patient lives. Specialist health care should cooperate with municipal services, and for the patient, the health care service should appear integrated. The plan also stated explicitly that large cities/towns should have ACT teams. ICT systems should also support good work processes and patient pathways.

The *National Health and Hospital Plan 2020-2023* (in Norwegian:* Nasjonal helse- og sykehusplan 2020-2023*) [8] was a revision of the *National Health and Hospital Plan 2016-2019*. The main goal of the new plan was to achieve a patient-centered care system in a sustainable way. The focus areas of the plan were better cooperation between specialist care and municipal services, improvement of mental care, focus on technology, improved digitalization, and to ensure health care workers with the right competence are available. It also underlined the need for cooperation between specialist health care and municipal health care. To improve patient pathways, the plan describes a need for ICT systems to share information between the different levels of health care. According to the plan, some of the most important measures to improve digitalization of the health care are to continue modernizing electronic health records (EHRs), improving access and availability of health information, and supporting digitalization of health and care services in the municipalities. The plan also promotes team-based methods of working, such as ACT and FACT.

#### Main Research Topic 3: Health Services Delivery Specific for Mental Health

##### Overview

The *Norwegian National Action Plan in Mental Health (1999-2008)* (in Norwegian: *Opptrappingsplan for psykisk helse 1999-2008*) [22] indicated several issues with the mental health care services. This included lack of prevention, poor services in the municipalities, often too short inpatient stays, and a lack of follow-up after discharge from a hospital. In this regard, the main goal of the plan was to strengthen mental health care and make more holistic and coherent care services available. It also pointed out that coordinated services were needed for persons with serious mental health services.

*Coping With Life: The Government’s Strategy for Good Mental Health (2017-2022)* (in Norwegian: *Mestre hele livet - Regjeringens strategi for god psykisk helse 2017-2022*) [9] was the first mental health strategy to follow The Norwegian National Action Plan in Mental Health (1999-2008). This document presented a holistic strategy for the field of mental health. The aims were to make mental health a part of public health work; promote inclusion into the society; focus on patient-centered care; improve knowledge, quality, research, and innovation in the services; and have a special focus on children and youth. The white paper also states that there is a need for research-based development of digital tools and guided internet-assisted treatment, and that experiences with ACT and FACT teams show that they lead to reductions in involuntary treatment. The paper also focuses on labor participation and housing, areas that are also important for FACT teams.

Based on the content identified from the policy documents that were deemed relevant, we defined the following themes of data: (1) eHealth solutions and infrastructure, (2) organizational management, and (3) health care services coordination.

The focus of the content included in each theme of data was the basis to further identify subthemes of data. The relevant content from the policy documents was then categorized under the subthemes of data, as presented below.

##### eHealth Solutions and Infrastructure

*Electronic communication* refers to ICT communication systems between health care institutions for the exchange of patient information [17].

*National digital services and ICT infrastructure* include digital services for citizens, process support that should be facilitated by the systems in use, and ICT infrastructure that should support the national deployment of eHealth [8,17,22].

*Data sharing and access* concern interoperability of systems in the sense of sharing of patient information between different levels and institutions in health care, and integration of ICT systems [8,17,20].

*eHealth records* refer to the requirement to update EHR systems to comply with current user needs [8].

*Secondary use of data* means the reuse of information for quality improvement, monitoring, management, and research [17].

##### Organizational Management

*Process support* identifies the need for ICT to support care pathways [21].

*Access to care* refers to health care services being provided to patients when and where needed [20-22].

*Care service delivery* concerns how patients receive health care services, including national patient pathways [8,20-22].

*Financing and prioritization of mental health care* refer to the national strategy for the field of mental health [20-22].

*Competence* refers to ensuring health care workers with the right competence are available [8,21].

*Prevention, early intervention, and follow-up* refer to municipalities taking responsibility for preventive care, early intervention, and follow-up [22].

##### Health Care Services Coordination

*Coordinated services* refer to the cooperation between different levels of health care, including health ICT development [8,21,22].

*Mental health services* indicate that mental health services should be coordinated so that patients receive care services from the appropriate level of care [9,20].

*Team-based health care services* refer to the use of team-based methods in health care, such as ACT and FACT [9].

The “eHealth solutions and infrastructure” theme is discussed in 4 policy documents, the “Organizational management” theme is also discussed in 4 policy documents, and the “Health care services coordination” theme is discussed in 5 documents.

### Laws and Regulations Governing eHealth

There are several Norwegian laws and regulations governing eHealth that are relevant for FACT teams. These include the Personal Data Act [23], the Patient Journal Law [24], the Health Personnel Law [25], and the Patient journal regulation [26].

The Personal Data Act applies the requirements of European Union’s General Data Protection Regulation (GDPR). The Personal Data Act and GDPR state that a data controller is the person or institution responsible for ensuring that the data are treated according to the principles relating to personal data processing. The data controller must establish the necessary technical and organizational measures to ensure that the laws are followed [27]. A data processor is a person or institution that processes data on behalf of a data controller [28]. Medical research on humans, human biological material, or health information needs to be preapproved by the Regional Committees for Medical and Health Research Ethics for the relevant region [29]. The committee does an appraisal of research ethics and if the project is in accordance with relevant laws.

The Patient Journal Law §19 allows for relevant and necessary information about a patient to be made available for health care workers when it is needed for providing, administering, or ensuring the quality of health care [24]. This is regardless of where the patient was treated earlier and how the health service is organized [21].

The Health Personnel Law §25 states that unless the patient refuses, cooperating health care workers can be given access to patient information when this is necessary to provide health care [25]. For 2 or more health care institutions to both have access and be able to update common EHR information, the Patient Journal Law §9 states that there needs to be a written agreement about how the institutions shall cooperate [24]. The Patient Journal Regulation [26] states that EHR information should only be available for health care workers who can confirm their identity in a secure manner.

The Norm gives institutions that follow it the necessary technical and organizational tools to ensure security and privacy when processing health information. The Norm includes several fact sheets and guides for processing health information. Some fact sheets that are relevant for FACT teams are for electronic messages, internal communication, and access to health information between organizations. A fact sheet from The Norm [30] specifies that to ensure security, data should be encrypted, authentication is necessary to access data, and access should be logged and monitored. To request sharing of data between institutions, a risk assessment is required for, and the partners who share information need to have an agreement in place about the data sharing. Patients have the right to see information about who has had access to their patient journal.

In practice, EHR data are usually easily accessible for health care workers in the institution that is responsible for the data, but harder to access for health care workers outside the institution. To circumvent this, health care workers who need access to patient information from an institution they are not affiliated to have sometimes been hired in a so-called zero percent position or simplified employment in the institution. This gives them access to the institution’s EHR, as employees of the institution.

### eHealth Infrastructure

#### Situation in Norway

Norway is divided into 4 health regions that are run by state-owned regional health authorities [31]. The regional health authorities are responsible for offering specialist health care to the population in the region. Each year, the Ministry of Health and Care Services gives the regional health authorities a commissioner’s document, which stands as a reference document concerning the needs for the health care sector in each region. How these needs are implemented are defined by each regional health authority. This means that the different regional health authorities have different plans for what eHealth solutions they will develop and use.

#### EHR Systems

The hospitals in 3 of the 4 health care regions are using the EHR named after the provider DIPS AS. The exception is the hospitals in the Central Norway Regional Health Authority, which are using DocuLive provided by Siemens AS. In the Norwegian primary care sector, there are several different EHR system vendors. However, the ongoing project Helseplattformen [32] is working on the implementation of a common EHR system for specialist and primary care in the central region of Norway.

For Norwegian FACT, which is organized as a cooperation between specialist mental health care and services from 1 or more municipalities, this means that the team members have different EHR systems available. Because of this, the teams must do the extra work of documenting in several EHRs or accept that some of the EHR systems will lack data.

#### Electronic Messages

In the Norwegian health care sector, standardized electronic messages are used to communicate between the different levels of care. Some of the types of messages sent are referrals, discharge letters, blood test results, and messages regarding sick leaves.

To ensure correct addressing for electronic and non-electronic communication in health care, there is a national address register for health care institutions. This allows for the standardized electronic messages to be sent from the sender’s EHR to the receiver through the Health Net [33]. Specialist care and municipal mental health services are among the partners who can use standardized electronic messaging. An evaluation of Norwegian FACT teams [7] showed that there are mixed experiences with standardized electronic messaging when used in the context of FACT teams. The report identified discharge letters from hospitals and documentation of medication as standardized electronic messages that were useful. By contrast, the report stated that messages from the outpatient clinic were too slow and identified this as a barrier for cooperation.

#### Videoconference

Several solutions have been used for video consultations with patients in Norway. The Norwegian national health portal Helsenorge [18] offers seamless integration of third-party videoconferencing solutions from their portal. Several different vendors have been approved for video integration. This means that there is no common solution for the use of videoconferencing for patient consultations in Norway. Even though FACT teams are moving toward the use of the video service provided by Helsenorge [18], at present, each FACT team can decide on how to implement and what videoconferencing solution to use following the guidelines of the health region they are in.

#### The Norwegian Summary Care Record

The SCR is a collection of health information for patients, which is available for all levels of health care in Norway. The goal of the SCR is to provide health care workers in different institutions quick access to important health information about patients. Patients have the possibility to choose not to have an SCR, and they can limit who has access and can deny access to parts of their SCR. The SCR can contain medication list, contact information, admission history, and critical information including a psychiatric emergency plan. Citizens can register information about their primary contact person, disease history, special needs, and information about being an organ donor. Somatic and mental health are treated equivalent in the SCR, and the admission list may also show visits to the psychiatric ward [34].

FACT team workers have access to the SCR like other health care workers and can use it to access medication lists and other relevant information.

### Relevant Literature on FACT Teams

Our searches returned 59 results in PsycINFO and 20 results in PubMed. We removed 10 duplicates, for a total of 69 results. After a review of the titles, 18 articles were selected for an abstract review, and 51 was excluded. In the abstract review, 3 articles were selected for a full-text review. These 3 articles matched our inclusion criteria. The 3 included articles describe eHealth interventions targeting ACT patients.

**Figure 1:**
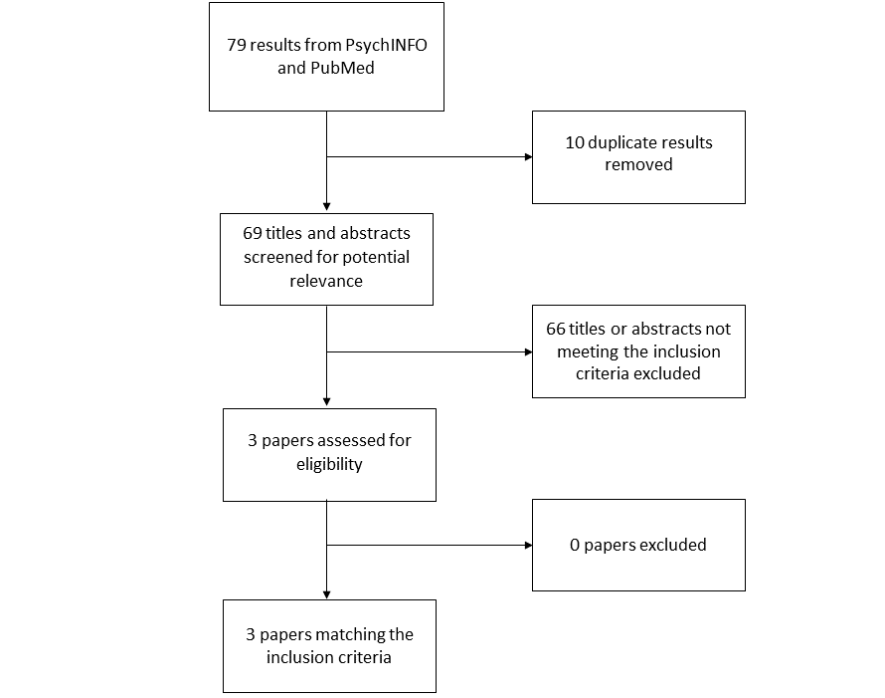
PRISMA flowchart of the literature search and article selection.

Figure 1 presents the results based on the Preferred Reporting Items for Systematic reviews and Meta-Analyses (PRISMA) guidelines [35].

Ben-Zeev et al [36] described a randomized control trial on texting mobile intervention added to ACT. In the trial, mental health workers had recovery-oriented texting exchanges based on the patients’ individual needs. The trial was feasible, acceptable, and safe. Looijmans et al [37] described a study protocol for a randomized controlled trial of a web tool intervention designed to improve cardiometabolic health in patients with severe mental disorders, including ACT patients. The intervention group received motivational interviews and a web tool that covers behavior awareness, lifestyle knowledge, motivation, and goal setting. Swanson and Trestman [4] described the use of a videoconference solution to supplement the face-to-face relationship with the team’s psychiatrist. The videoconference solution is used for crisis intervention and for augmentation of the established face-to-face treatment. The solution has been accepted by the staff and the patients, and seriously ill patients have been able to use the solution. The solution has also reduced travel time for the psychiatrists.

### FACT Implementation

*The FACT Handbook* [3] is a description of the FACT model written by one of the founders of the model. It was commissioned by the Norwegian Directorate of Health and has been used as a guide for implementing new FACT teams in Norway.

A report on the evaluation of Norwegian FACT teams [7] showed that all 7 teams evaluated used electronic whiteboards to display patient information. Some teams had bought a commercial solution for a whiteboard, while others had developed them locally or used the solutions from other teams. The report also demonstrated that there are different approaches to using EHRs in Norwegian FACT teams. Two of the 7 teams document in 2 separate EHRs: 1 for primary care and 1 for specialist care. However, 5 teams use the EHR only for specialist care. In these FACT teams, team members who work in primary care are given “zero percent positions” in the specialist care. This means that they are formally hired, to allow access to the specialist health care EHR. Interviews conducted with the FACT teams’ cooperating partners also emphasize the challenges of working with different EHR systems. Some cooperating partners found use of standard electronic messages useful for improving coordination. Others found that the sent standard electronic messages took too long before they were received, and that this did hinder the ability to provide integrated services to the patients. The report also pointed out that different regulations governing FACT teams and municipalities and the multiple EHR systems used by the teams hindered service integration. One of the main recommendations of the report is that digital communication solutions should be facilitated to improve coordination.

A study on how Norwegian FACT teams are integrated into the Norwegian health care system [10] showed that FACT teams reduce complexity and reassure other services by taking responsibilities for treatment and follow-up of patients. However, the study also showed that there is a lack of common communication systems, making exchange of information harder and more time consuming. The study also reported that not all patient information is available in the different EHR systems in use.

## Discussion

### Norwegian Policy Regulating Mental Health Services

Policy documents identify ACT and FACT teams as a wanted part of the Norwegian health care system. ACT and FACT have been recommended in the policy documents *Coping With Life: The Government’s Strategy for Good Mental Health (2017-2022)* [9], the *National Health and Hospital Plan 2016-2019* [21], and the *National Health and Hospital Plan 2020-2023* [8].

Considering the number of policy documents targeting each theme of data, we conclude that the 3 themes of data considered are relevant for the implementation of FACT teams in Norway.

#### eHealth Solutions and Infrastructure

Policy documents state that better eHealth solutions are needed in health care. Examples of this are the *One Citizen–One Journal* [17], which stated that the goal of the government is to make electronic communication as the standard way of written communication within health care; and the *National Health and Hospital Plan 2016-2019* [21], which states that ICT systems should also support good work processes and patient pathways. The *National Health and Hospital Plan 2020-2023* [8] renews this vision by stating that ICT systems are needed to share information between the different levels of health care. The plan also states that further modernization of EHRs is needed.

While the policy documents do not specifically discuss ICT challenges for FACT teams, these issues are also present in FACT teams, with the lack of horizontal and vertical collaboration in health care. This is mainly seen in the lack of electronic communication and sharing of patient information between the team members. The needs from FACT teams are the same as those described in the policy documents: National digital services and ICT infrastructure. The implementation of the work processes defined by the FACT model is not supported by the existing eHealth system. For them to work as expected, there should be similar implementation of the FACT model across the country. Besides, the ICT infrastructure needs to evolve to comply with the requirements of the FACT model, which implies the deployment of national digital services for FACT teams. Secondary use of health data relevant for FACT teams, such as quality improvement, monitoring, management, and research, will also be hindered until better eHealth systems are in place.

#### Organizational Management

One vital aspect of the policy documents is describing the organization of the health care service. Three of the identified strategy documents [20-22] state that the field of mental health should be prioritized. Four documents also state that ACT or FACT teams should be implemented [8,9,20,21]. Four of the 5 identified strategy documents refer to access to care and care service delivery, with an emphasis on implementing standardized patient pathways [8,20-22]. In this context, 1 policy document points to the lack of follow-up after hospital discharge as an issue [22]. One of the primary goals of FACT teams is to improve follow-up for their patient group.

The essence of the FACT model is the delivery of integrated care through multidisciplinary teams. As emphasized in the policy documents, FACT teams also need to have access to the required competence to be able to provide integrated care services with the expected quality. In practice, FACT teams must make work-arounds to deliver integrated care services as the existing ICT infrastructure does not facilitate process support, as mentioned in the policy documents.

#### Health Care Services Coordination

Even though team-based methods of care delivery, such as ACT and FACT, are promoted [8], the lack of coordination among the Norwegian health care services is one of the main challenges of the sector at present. This has been described in general for the health care sector in the *Coordination Reform* [20], as well as in the policy documents specific for mental health: *The Norwegian National Action Plan in Mental Health (1999-2008)* [22] and *Coping With Life: The Government’s Strategy for Good Mental Health (2017-2022)* [9]. The lack of coordination between the different levels of care and between institutions greatly affects the way FACT teams are implemented, as the cornerstone of the FACT model is the delivery of coordinated services.

FACT teams in Norway have found ways to overcome the lack of coordination in the health care sector to still be able to deliver coordinated services. This includes the aforementioned “zero percent positions,” which allow all team members access to EHR information from specialist care. The evidence that FACT teams provide better coordinated services and contribute to the reduction of both emergency admissions and forced admissions has resulted in the implementation of several FACT teams in Norway [7].

### eHealth Regulations

One barrier for FACT teams is eHealth regulations. However, the Patient Journal Law §19 allows necessary information about a patient to be made available for health care workers when it is needed for providing, administrating, or ensuring the quality of health care. Besides, the Health Personnel Law §25 states that health information can be given to cooperating health care workers when this is necessary to provide health care. However, in practice it is often hard for health care workers to access EHR data from institution where they do not work. This shows that current ICT solutions do not take advantage of what is made possible by the laws and there is a need for systems to allow EHR access to relevant data, while preserving the privacy of the information.

In the context of the personal data law and GDPR, hospitals or health trusts take the role of data controllers, responsible for EHR data in their areas. EHR vendors are data processors that process data on behalf of the controllers. There is a need for an agreement between the data controller and data processor, stating the obligations of each of the partners.

In addition to the laws, the Norm is a helpful guideline when implementing ICT solutions in Norway, which provides necessary information for practical implementation. The Norm also specifies responsibilities of data controllers and data processors.

### eHealth Infrastructure

Lack of integration and coordination between services and technologies has been seen as an issue for use of eHealth within psychiatry [38]. Also in Norway, implementation of FACT teams has been affected by the eHealth infrastructure. Sharing of data has been reported as a problem for Norwegian FACT teams [7]. The specialist and primary care in Norway use different EHR systems, and the technical infrastructure in place does not allow easy exchange of information between systems. This leads to challenges for FACT teams that often have members from both specialist and primary health care.

Different health regions in Norway can also have different implementation of regulations. There are various ways of bypassing these challenges, but ideally the EHR systems should be able to display relevant EHR data to the health care workers who need the information for treating their patients, even when the information is stored in different systems. In general, there are challenges with sharing information between the different levels of health care and different regions on the same level. Use of electronic messages is one well-established way of doing this and is useful in many contexts. However, the use is still limited to standard messages, such as referrals and discharge letters.

Videoconferencing has been used for both communication between health care workers and communicating with patients. The COVID-19 pandemic increased its use in many FACT teams. Video consultations with patients might be a useful tool for FACT teams, but it might not be suited to all their patients. Various solutions for video consultations are used in Norway, and some of these have integration to the portal Helsenorge [18] and the calendar in DIPS.

The only data that are shared with all health care workers in Norway are the SCR. This record includes information on medication, which can be useful for FACT teams. However, the intention of the SCR is not to support clinical cooperation.

### Relevant Literature on FACT Teams

Our literature search returned 3 articles that matched our inclusion criteria, which reported on the use of an SMS text message intervention [36], a web tool [37], and the use of videoconferencing [4]. These 3 articles showed that eHealth interventions can successfully target ACT patients. However, we found no articles discussing ICT challenges for ACT or FACT teams themselves. While we did not perform a full literature review, this implies that there is little or no research on this topic. Health care is organized differently across countries, and many of the challenges regarding eHealth infrastructure and regulations described in this article are not relevant in other countries. Thus, the lack of articles on this topic is not surprising.

### FACT Implementation

The evaluation of FACT teams has shown their positive effects on patients, including reduction of inpatient days, with a larger reduction in compulsory inpatient days [7]. The evaluation also highlights some of the ICT challenges the teams face, including EHR access. A study of how FACT teams fit into the Norwegian service model also showed that the cooperating partners of FACT teams think the lack of common communication systems and EHR systems is a challenge [10]. These findings show that, despite some issues with EHR systems, the implementation of FACT teams in Norway has been successful and can be expected to continue.

### Conclusions

Weaknesses in the Norwegian eHealth infrastructure have been a barrier for an easy implementation of FACT teams in Norway. The FACT evaluation report identifies the sharing of information between the different levels of health care as a main shortcoming of the existing eHealth infrastructure. FACT teams need eHealth systems that allow easy and secure sharing of health information with the team members and other relevant health care workers to provide better care. There is also a lack of research on the ICT challenges facing FACT teams. This means that there is a need for research studying the eHealth challenges and needs of FACT teams in greater detail. Furthermore, there is a need to explore how eHealth solutions should be designed to support FACT teams in a Norwegian context. This is something we will focus on in future work.

## Abbreviations

ACT: Assertive Community Treatment
EHR: electronic health record
FACT: Flexible Assertive Community Treatment
GDPR: General Data Protection Regulation
ICT: information and communications technology
NHN: Norwegian Health Net
PRISMA: Preferred Reporting Items for Systematic Reviews and Meta-Analyses
SCR: summary care record
